# Child vs. Parent

**Published:** 1879-04

**Authors:** 


### CHILD vs. PARENT.

UNCLE BOB.

There is in every form of life, whether of beast or man, a law of self preservation which leads the weaker to oppose all its strength to the endeavors of the stronger to overcome it. We know that the first faculties developed are simply animal in character,—"instinct." With the brutes, we can develop nothing beyond. They learn by experience that force of one form or another will compel subjection. The process by which this is accomplished, rendering them useful for our domestic service, we term "breaking in."

The earliest management of the human species is in one sense akin to this, but a sense of wrong is innate, therefore justice, not brute force, is essential to the growth of good government in establishing the family relations. Whether in the brute creation there are more than the mere shadowings of the same truth, is not within our purpose at present. Every child, from almost the first moment of its birth, sets up and asserts a will, independent and as self-confident of success in its little purposes as in the greater ones of its maturer years.

The case of "Child versus Parent" is opened. The arguments are loud and forcefully urged. For a period, reason has nothing to do in the contest, but as days pass on to the time when the faculties show their development, the thoughtful parent sees a necessity for exercising the greatest care in developing in the child a spirit of ready acquiescence to enforced authority. The very fact that feebleness is opposed to might, makes an occasion for the most careful exhibition of the parent's will. How easily in the vexations and worry of numberless cares, is it for the parent to compel by force what would otherwise have been accomplished by the employment of milder means.

We hear a great deal about vicious inclinations of children. They are largely the growth of errors in management and education,—a result of either over-severity or indulgence—the final result being the "spoiled child." Either the affections have been alienated or duty has been lost in selfish petulance. Which of the two is preferable is an open question as between child and parent. A self reliance, engendered of a successful accomplishment of its aims will, perhaps, make a better citizen of the former, but the peace of the family is gone in either case.

The use of punishment for securing obedience must be decided most wisely. Many a time doubt will arise as to the right or wrong of conduct. The governing motive is to be sought out, whether it was ignorance, thoughtlessness, or the desire for having its own way. If a parent finds a perplexity as to the decision to be reached, does not that show a doubt as to the rights and wrongs in the matter—for otherwise how clear would be the judgment. Study it well—the good to yourself, and the child are worthy of hesitation before action. When your course is made clear, be perfect in enforcing your correction. The certainty that punishment follows disobedience is of greatest value. The time for argument has passed, and respect or disrespect for your wisdom is the result.

Do not promise or threaten and leave to chance its fulfillment, otherwise there will be a premium placed upon the possibility of escaping again. A tyrant on his throne may be preferable to a weakling, who vacillates 'till authority is never dreamed of in his dominion. Fire *always* burns, and water *always* drowns those who disregard its inevitable laws.

As the years ripen and the developement of the body brings additional powers, the field for the excercise of parental influence affords the parent more abundant resourses for accomplishing the very best results. It is, however, at a period before the child is twelve years of age, that character for good or evil has been formed in the majority of our children. The parents authority and constant watchfulness have been superseded by the society of playmates and the discipline of teachers at school. If family government has not already excercised a restraining influence upon the natural desires, it is doubtful whether the child can overcome the temptations of its new experiences. Have a care, then, for your child while you have it entirely under your influence. Be sure that you are respected, loved, and recognized as supreme authority, having the right and wisdom to decide all doubts, so making straight the path of duty for all your dependant ones.

